# Qualitative and quantitative analysis of the bioactive components of “ginseng–polygala” drug pair against PC12 cell injury based on UHPLC-QTOF-MS and HPLC

**DOI:** 10.3389/fphar.2022.949757

**Published:** 2022-12-09

**Authors:** Zhengyang Wang, Xiaomeng Sun, Yue Zhao, Liba Ga, Qi Li, Qian Li, Xiaotong Wang, Chunjuan Yang

**Affiliations:** Department of Pharmaceutical Analysis and Analytical Chemistry, College of Pharmacy, Harbin Medical University, Harbin, China

**Keywords:** “ginseng–polygala” drug pair, high-resolution mass spectrometry, structural identification, quality control, data processing

## Abstract

Aβ_25-35_-induced PC12 cells were used as the *in vitro* injury model to evaluate the effects on PC12 cells after intervention with the “ginseng–polygala” drug pair. The results showed that the drug pair could significantly increase cell activity and reduce the level of reactive oxygen species and the concentration of inflammatory factors to improve the Alzheimer’s disease treatment process. Furthermore, to rapidly identify and classify complicated bioactive components of the drug pair, a liquid chromatography with quadrupole time-of-flight mass spectrometry method combined with a molecular network strategy was established. With this strategy, 40 constituents were preliminarily identified and a database of the compounds was successfully established. Among them, 12 compounds of different categories were accurately identified by comparison with reference substances. The content of the aforementioned active components was simultaneously determined by HPLC to control the quality of compatible medicinal materials, and the verification results of the analytical method met the content determination requirements. The results revealed that after compatibility, the content change of the components is not the simple addition of quantity but the comprehensive effect of the two medicines. In conclusion, this study could provide a generally applicable strategy for pharmacological activity, structural identification, and content determination in traditional Chinese medicine and its compatibility.

## 1 Introduction

Alzheimer’s disease (AD), also known as senile dementia, is a degenerative disease of the central nervous system, whose etiology is still unclear (Soria Lopez et al., 2019). According to statistics, the number of elderly people with AD in China has exceeded six million, and it is expected that the number will exceed 20 million by 2050. With the worsening of the aging society, the number of patients is increasing. AD has become one of the major health and socio-economic problems facing the world (Yasuno et al., 2020; Nielsen and Boenink, 2021). Therefore, prevention and treatment of AD and the research and development of anti-AD drugs are particularly important and urgent. At present, most of the drugs used in clinical treatment of AD are chemical agents. Although effective, it only alleviates some of the symptoms of the disease process (Se Thoe et al., 2021). However, traditional Chinese medicine (TCM) treatment has the characteristics of overall effect and synergistic effect, which can improve the disease process through a variety of ways to meet the treatment needs of the complex pathological mechanism of AD. (Deng et al., 2020; Fang et al., 2020; Rahman et al., 2022). The curative effect of TCM is comprehensive and lasting, which has great advantages and potential, and is considered a promising research method (Bhattacharya et al., 2022).

According to ancient Chinese medicine records, Polygalae Radix (the dried root of *Polygala tenuifolia* Willd or *Polygala sibirica L.* *,* PR) and Ginseng Radix et Rhizoma (the dried root of *Panax ginseng* C. A. Mey., GR) are mainly used in the treatment of amnesia dementia (Zhao et al., 2017; Xu and Zheng, 2021). Furthermore, according to “Shennong Classic of Materia Medica” and “Compendium of Materia Medica”, GR and PR are often used in combination in TCM prescriptions. The application of GR could be traced back to about 5000 years ago, thanks to its various beneficial biological activities (Mancuso and Santangelo, 2017; Razgonova et al., 2019), and PR has also been verified to be applied with GR to exhibit protective effects on the central nervous system and was frequently used to treat memory dysfunction, insomnia, and neurasthenia (Deng et al., 2020; Liang et al., 2021; Hussien and Yousef, 2022). Therefore, the combination of GR and PR as a drug pair (GR + PR) may have an optimistic application prospect in the treatment of AD. However, changes in chemical components and contents often occur during the compatibility process, which may affect the performance of its efficacy. Consequently, it is very necessary to carry out pharmacodynamics research, composition characterization, and quality control of GR + PR.

Most of the current studies focus on the therapeutic effect of GR, PR, or a certain active ingredient to research the pharmacological effects (Rajabian et al., 2019; Wang et al., 2020; Zhang et al., 2020). At the same time, there are many qualitative determination methods for GR or PR; for instance, Yang et al. identified the representative compounds and characteristic markers of GR by ultra-performance liquid chromatography quadrupole/time of flight-mass spectrometry; and this analysis method was also used by Li et al. to rapidly characterize compounds in PR (Wu et al., 2015; Yang et al., 2015; Zhu et al., 2018; Li T. et al., 2021; Yang et al., 2021). It can be seen that the current research mainly focuses on the efficacy and composition of a single drug and lack of comprehensive research on the drug pair. Moreover, the molecular networking (MN) strategy has the characteristic of clustering the same type of chemical components, and its combination with high-resolution mass spectrometry would be beneficial to intuitively clarify the material basis of TCM (Quinn et al., 2017; Liu et al., 2021). Therefore, we proposed a systematic strategy for drug pair research, which considered the synergistic application of GR and PR to explore whether the change of composition and content would affect its efficacy.

In this study, first of all, an PC12 cell injury model induced by Aβ_25-35_ was used to evaluate GR + PR in cell activity, oxidative stress, and inflammatory factor aspect pharmacodynamics *in vitro*. Then, on the premise of clarifying the pharmacological effect, the active components that could play a role in GR + PR were explored. The pharmacodynamic substance composition of GR + PR can be better characterized through combining the MN strategy with UHPLC-QTOF-MS and is conducive to elucidating the synergistic effect of active components. Also, this strategy would also bring a new way for the study of the modernization of TCM, whereafter, the content of main active ingredients in GR, PR, and GR + PR was determined by typical HPLC, and the influence of the compatibility on the composition and content was explored while controlling the quality of the drug pair. This research provides a systematic strategy for comprehensive evaluation of the pharmacological effects, chemical composition, and quality of GR + PR. Furthermore, it is expected that the methods and results of this study could provide a useful methodological and chemical basis for further study of GR + PR and other TCM prescriptions.

## 2 Materials and methods

### 2.1 Materials and reagents

The PC12 cells, TNF-α, IL-1β, and reactive oxygen species (ROS) were obtained from Boster Biological Technology (Wuhan, China), Aβ_25–35_ was obtained from Sigma-Aldrich (St Louis, MO, United States). MTT was purchased from Beyotime Biotechnology (Shanghai, China). DMEM High Glucose was purchased from HyClone (UT, United States). Fetal bovine serum and penicillin/streptomycin were purchased from Gibco (CA, United States). Dimethyl sulfoxide was purchased from Amresco (NJ, United States). Acetonitrile and methanol were of HPLC grade (Tedia). Formic acid was of HPLC grade (Tianjin, China). Distilled water was provided by the Wahaha Company (Hangzhou, China). Sibiricose A5 (A5), sibiricose A6 (A6), sibiricaxanthone B (SB), tenuifoliside B (TenB), 3,6′-O-diosinoyl sucrose (3,6′-O-ds), tenuifoliside A (TenA), ginsenoside Re (GRe), ginsenoside Rb1 (GRb), ginsenoside Ro (GRo), ginsenoside Rf (GRf), ginsenoside Rd (GRd), and tenuifolin (Ten) standards were provided by Chengdu Preferred Biological Technology (Sichuan, China), the purities of all reference compounds were above 98%. *Panax ginseng* and *Polygala tenuifolia* herbs were obtained from Tongrentang (Beijing, China). The automatic microplate reader was purchased from Thermo Fisher (MA, United States). The UHPLC-QTOF-MS system was performed with an Agilent 1260 UHPLC system and a triple TOF 5600 mass spectrometer (AB SCIEX, Foster City, CA). HPLC-2030plus was purchased from Shimadzu (Japan). RE-52A Rotary Evaporators were obtained from Yarong (Shanghai, China). The AE240 Electronic Balance was obtained from Mettler (Germany).

### 2.2 Sample and standard solution preparation

The same amount of ginseng and polygala powder was extracted by sonication triplicate with methanol for 60 min, and the working solution of the drug pair was prepared. In addition, the extraction method of ginseng and polygala is the same as previously mentioned. Prior to the injection and analysis by HPLC, all extracts were passed through 0.45-µm nylon membranes.

Stock of A5, A6, SB, TenB, 3,6′-O-ds, TenA, GRe, GRb1, GRo, GRf, GRd, and Ten standard solution was prepared by accurately weighing standard 4.00 mg and dissolved in 1.0 ml methanol. Working standard solutions were obtained by appropriate dilution of the stock solution with methanol.

### 2.3 Cell culture and detection methods

#### 2.3.1 Cell culture

The PC12 cells were cultured in H-DMEM supplemented with 10% FBS and 1% penicillin/streptomycin. The cells were incubated at 37°C in moist air containing 5% CO_2_ (Yang et al., 2019; Wiatrak et al., 2020). PC12 cells were seeded in culture plates (1×10^4^ cells/ml) for 24 h and then treated with a series of concentrations of GR, PR, and GR + PR for 24 h. Subsequently, Aβ_25-35_ was added to the medium and co-cultured with the drugs for an additional 24 h. The PC12 cells were divided into five groups, namely, the control group, Aβ group, GR group, PR group, and GR + PR group.

#### 2.3.2 Cell viability test

The cells were seeded in a 96-well tissue culture plate at a density 1×10^4^ cells/well. The PC12 cells were incubated with varying concentrations of GR, PR, and GR + PR (2.5, 5, 10, 20, and 40 μg/ml) for 24 h. The PC12 cells served as the control group. After treatment, MTT was added to each well at a final concentration of 1 mg/ml, and the cells were further incubated at 37°C for 4 h. Dimethyl sulfoxide (150 μl) was added to each well after removing the medium. After shaking the plates for 5 min, the absorbance of the mixture was measured at 490 nm. Cell viability was expressed as the standard deviation, with 100% in the control group. Data were analyzed by one-way analysis of variance (ANOVA) using SPSS 19.0 statistical software. A test of homogeneity of variance was used with a significance level of 0.05.

#### 2.3.3 Detection of reactive oxygen species

Each sample with 20 μM DCFH-DA was added to the medium and incubated at 37°C for 60 min. After trypsin digestion, the cells were harvested by centrifugation at 1,200 rpm for 10 min, washed twice with PBS, and cell precipitate was collected by centrifugation for fluorescence assays. The collected cells were resuspended in PBS and detected by light avoidance. The excitation wavelength was 500 nm, and the emission wavelength was 525 nm, detected using a multifunctional microplate reader and recorded (Cheignon et al., 2017).

#### 2.3.4 Enzyme-linked immunosorbent assay

Enzyme-linked immunosorbent assay (ELISA) was used to detect inflammatory factors such as TNF-α and IL-1β. After treatment with cell administration, the supernatant medium was centrifuged at 1500 rpm for 10 min. The measurement was performed according to specific operation steps of enzyme-linked immunosorption and referring to previous reports (Li et al., 2017; Kohl and Ascoli, 2017; Hornbeck, 2015).

### 2.4 Qualitative analysis method by UHPLC-QTOF-MS

The UHPLC system consisted of a quaternary pump, an on-line degasser, and a column temperature controller. The chromatographic separation was performed on a Phenomenex Kinetex XB C18 column (100 mm × 4.6 mm, 2.6 μm) at 30°C. Analysis was completed with a gradient elution of 0.1% formic acid in water (A)/0.1% formic acid in acetonitrile (B) within 35 min. The gradient program was 95%–70% A from 0 to 13 min, 70%–60% A from 13 to 27 min, 60%–30% A from 27 to 30 min, 30%–10% A from 30 to 31 min, and 95% A from 31 to 35 min at a flow rate of 0.5 ml/min with a sample injection volume of 5 μl.

The Agilent 1260 UHPLC system was connected to a Triple TOF 5600 (AB SCIEX, Foster City, CA) with a negative ESI. The optimized parameters of MS conditions were as follows: ion spray voltage, −4500 V; declustering potential, 80 V; the turbo spray temperature, 550°C; nebulizer gas (Gas 1), 50 psi; heater gas (Gas 2), 50 psi; and curtain gas, 30 psi. Nitrogen (N_2_) was kept as a nebulizer, auxiliary, and collision gas. The TOF-MS scan was operated with the mass range of *m/z* 100–2000. A typical information-dependent acquisition was used to carry out the MS/MS experiment. For information-dependent acquisition, a sweeping collision energy setting at 60 ± 15 V was applied for collision-induced dissociation. Collision gas was set at 15 psi.

### 2.5 Quantitative analysis method by HPLC

The chromatographic separation was performed on a ZORBAX SB-C18 column (250 mm × 4.6 mm, 5 μm) at 30°C. Analysis was completed with a gradient elution of acetonitrile (A)/0.1% formic acid in water (B) within 100 min. The gradient program was 8%–13% A from 0 to 15 min, 13%–16% A from 15 to 16 min, 16%–18% A from 16 to 30 min, 18%–21% A from 30 to 31 min, 21%–22% A from 31 to 40 min, 22%–25% A from 40 to 50 min, 25%–28% A from 50 to 60 min, 28%–30% A from 60 to 67 min, 30%–37% A from 67 to 73 min, 37% A from 73 to 85, and 37%–38% A from 85 to 100 min at a flow rate of 0.9 ml/min with a sample injection volume of 20 μl. Also, the detection wavelength was 203 nm.

### 2.6 Data processing

First, a local database was established and all components were structurally classified to summarize the fragmentation patterns and diagnostic ions through AnalystTF v1.6 (Peakviewv2.2, Masterview™ v1.1) processing software. In addition, the molecular network map was established based on the secondary fragment similarity, and combining with the precise molecular weight, retention time, and secondary fragments, the structurally similar compounds were speculated.

## 3 Results and discussion

### 3.1 Pharmacodynamics research

**FIGURE 1:**
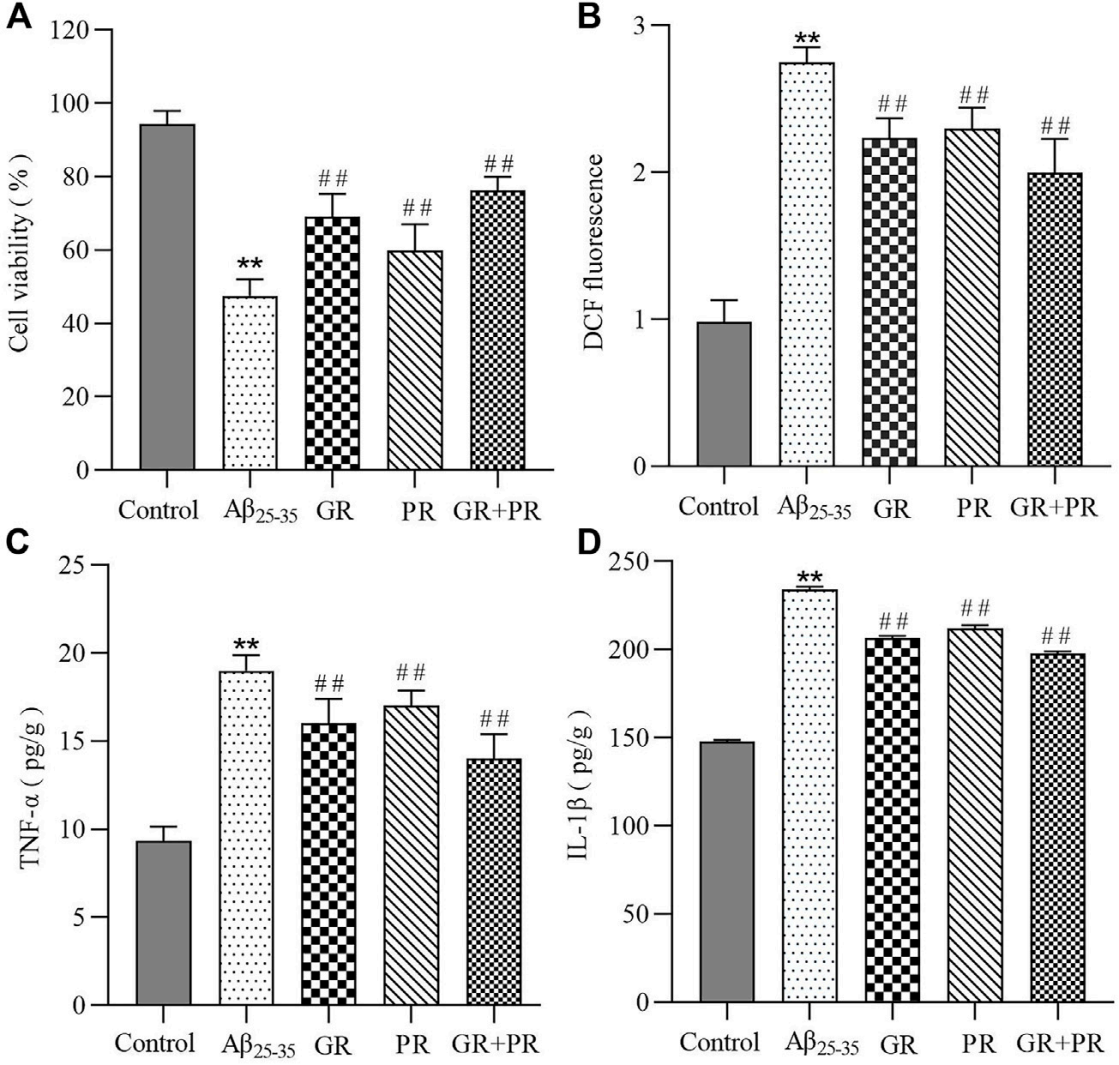
*In vitro* therapeutic efficiency of GR, PR, and GR + PR on Aβ_25-35_-induced PC12 cells. Cell viability **(A)**, ROS reduction **(B)**,and the inhibition of inflammation revealed by an alleviation of TNF-α **(C)** and IL-1β **(D)** on Aβ_25-35_-induced PC12 cells. Data represent the mean ± SD from six replicates. Compared with the control group, **p<0.01. Compared with the Aβ_25-35_ group, ^##^ *p* < 0.01.

To investigate whether GR + PR has a positive effect on AD, in this study, the PC12 cell model induced by Aβ_25-35_ was constructed initially. Then, the *in vitro* pharmacodynamics of GR, PR, and GR + PR with respect to cellular activity, proinflammatory factors, and oxidative stress levels were investigated. The MTT assay results revealed that the cell activity of GR + PR is significantly increased compared with that of GR or PR, as shown in Figure 1A. Moreover, it was found that in the PC12 cell model, the levels of TNF-α, IL-1β, and ROS were significantly increased. After administration of GR, PR, and GR + PR, the abnormal increase of TNF-α, IL-1β, and ROS could be inhibited. Also, GR + PR has the strongest inhibitory effect; therefore, the effect after compatibility was more significant. The results of ROS levels and proinflammatory factors are shown in Figures 1B–D.

These results indicated that GR, PR, and GR + PR had a positive effect on the abnormal change of cell activity, proinflammatory factors, and oxidative stress levels. However, GR + PR shows a better therapeutic effect than GR or PR. The reason may be that GR and PR have a synergistic effect after the combination, which can improve the cell activity by inhibiting the inflammatory response and the level of ROS. Multiple studies have reported that various oxidative stress factors, including lipid peroxidation, protein peroxidation, and DNA and RNA peroxidation, occur in the brain of Alzheimer’s disease (AD) patients (Islam, 2017). Simultaneously, both aggregated Aβ and hyperphosphorylated Tau protein could cause the activation of astrocytes during AD. Also, further release of proinflammatory factors, including TNF-α and IL-1β, causes nerve cell damage and neuroinflammatory response. The persistent inflammatory response can cause cognitive dysfunction and accelerate the pathological process of AD (Hur et al., 2020). Therefore, GR + PR may have a therapeutic effect on AD by improving the activity of PC12 cells and inhibiting neuroinflammation and oxidative stress.

### 3.2 Characterization of the chemical constituents of “ginseng–polygala” drug pair

In order to uncover the compounds of “GR + PR,” which may have a positive effect in AD treatment, a UHPLC-QTOF-MS analysis was conducted to summarize the fragmentation pattern and diagnostic ion. Then, the Molecular Networking (MN) technique, which is mainly based on the similarities and differences of secondary mass spectra between related nodes to infer structurally similar compounds, was used to quickly screen and identify the representative compounds in GR + PR. Finally, the main chemical components of GR + PR were characterized and identified based on UHPLC-QTOF-MS analysis and MN strategy.

#### 3.2.1 Mass spectrometry fragmentation pattern and diagnostic ion analysis

Many studies show that GR + PR mainly include ginsenosides, polygala saponins, oligosaccharide esters, and xanthones (Yang et al., 2019; Sun et al., 2020). According to the structure of the nucleus, ginsenosides are mainly divided into oleanane-type (OLE-type) pentacyclic triterpenes and dammarane-type tetracyclic triterpenes, which could also be divided into protopanaxadiol type (PPD-type) and protopanaxatriol type (PPT-type) based on the presence of hydroxyl in the 6 position of carbon (Shi et al., 2019; Hou et al., 2021). Polygala saponins are mainly oleanane-type pentacyclic triterpenoid saponins. Polygala oligosaccharide esters are composed by connecting different phenyl acrylic acid groups and sugar groups. Polygala xanthones exist in plants in the form of glycosides, including structures connected by O-glycosidic bonds and C-glycosidic bonds (Klein et al., 2012; Cheng et al., 2021).

**FIGURE 2:**
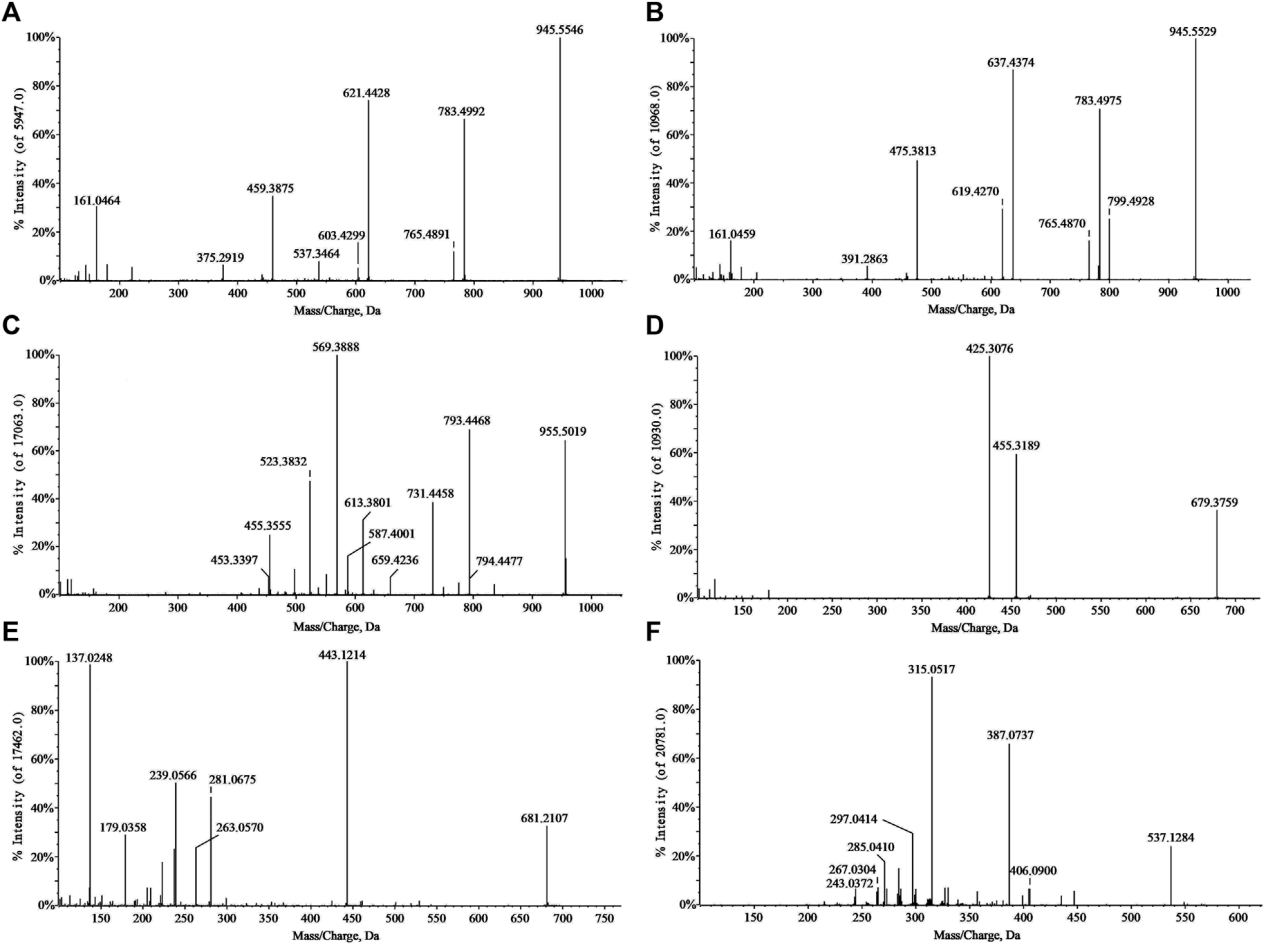
MS/MS spectra of standard substances. GRd **(A)**; GRe **(B)**; GRo **(C)**; Ten **(D)**; TenA **(E)**; and SB **(F)**.

**FIGURE 3:**
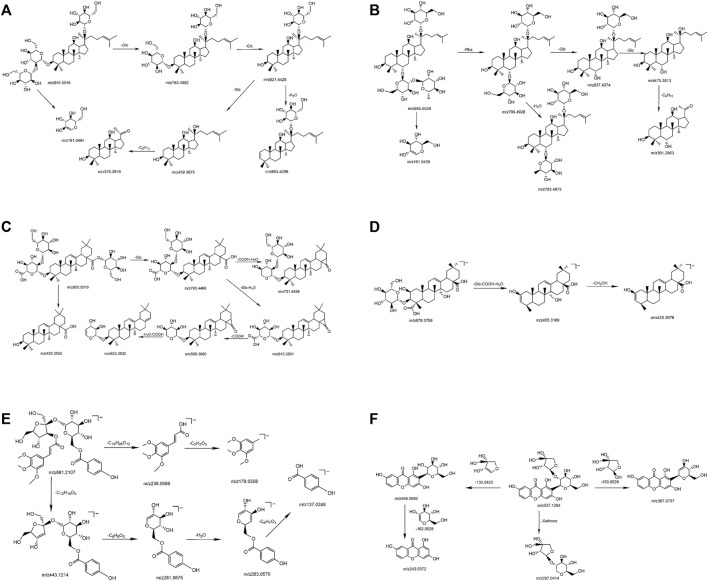
The possible cleavage pathway of typical compounds. GRd **(A)**; GRe **(B)**; GRo **(C)**; Ten **(D)**; TenA **(E)**; and SB **(F)**.

In the negative ion mode, standards solutions of the aforementioned six types of representative compounds (GRd, GRe, GRo, Ten, TenA, and SB) were analyzed by high-resolution mass spectrometry to determine the characteristic fragment ions, typical neutral loss molecules, and possible fragmentation patterns. Also, the possible cleavage patterns were preliminarily explained combined with the cleavage information of MS/MS spectra in Figure 2, and then the cleavage pathways of the typical compounds are shown in Figure 3. For example, according to the MS/MS mass spectrum of GRd (Figure 2A), the excimer ion peak was *m/z* 945.5546. After collision-induced dissociation (CID), *m/z* 783.4992 was generated for loss of neutral glucose residues. Also, the fragment ions 621.4428 and *m/z* 459.3875 were produced by the loss of two and three molecules of glucose residues in the excimer ion peak, respectively. The possible cleavage pathway of GRd is shown in Figure 3A. According to the MS/MS mass spectrum of GRe (Figure 2B), the excimer ion peak was *m/z* 945.5529. After CID, characteristic ion fragments such as *m/z* 799.4928, 637.4374, and 475.3813 were generated. Thereinto, *m/z* 799.4928 was produced by the loss of neutral rhamnose residues, *m/z* 637.4374 was produced by the loss of rhamnose residues and the neutral glucose residues, and fragment ion *m/z* 475.3813 was produced by the loss of one molecule of rhamnose and two molecules of glucose in the excimer ion peak. The possible cleavage pathway of GRe is shown in Figure 3B. According to MS/MS mass spectrum of GRo (Figure 2C), the excimer ion peak was *m/z* 955.5019. After CID, characteristic ion fragments such as *m/z* 793.4468, 731.4458, 613.3801, 569.3880, 523.3813, and 455.3555 were generated. Thereinto, *m/z* 793.4468 was generated by the loss of one molecule of glucose, *m/z* 731.4458 was produced by the loss of one molecule of H_2_O and one molecule of COOH, *m/z* 613.3801 was produced by the loss of two molecules of glucose and one molecule of H_2_O, and *m/z* 569.3880 was produced by *m/z* 613.3801 losing one molecule of COOH. Then, after losing one molecule of H_2_O and one molecule of COOH, the fragment ion of *m/z* 523.3813 was generated. It should be noted that *m/z* 455.3555 is the characteristic fragment ion of the oleanane core. The possible cleavage pathway of GRo is shown in Figure 3C. As shown in the TOF MS/MS mass spectrum, PPD-type, PPT-type, and OLE-type ginsenosides have similar characteristic neutral loss, such as characteristic neutral loss 146.0579 and 162.0528. Polygala saponins are mostly triterpenoid saponins; among them, the representative compound Ten was taken as an example. According to the MS/MS mass spectrum of Ten (Figure 2D), the excimer ion peak was *m/z* 679.3759. After CID, the glycoside bond was cleaved, and a molecule of COOH was lost to generate fragment ion with a higher response of *m/z* 455.3189. Then, *m/z* 425.3076 was produced by further losing a molecule of CH_2_OH on this basis. The possible cleavage pathway of Ten is shown in Figure 3D. As shown in the TOF MS/MS mass spectrum, the main cleavage pathway of polygala saponins was the neutral loss of sugar residues, including 146.0579 and 162.0528. Polygala oligosaccharide esters are mainly composed by connecting different phenyl acrylic acid groups and sugar groups. According to MS/MS mass spectrum of TenA (Figure 2E), the excimer ion peak was *m/z* 681.2107. In the structure of TenA, the glucose group and the fructose group are, respectively, connected to a molecule of the p-hydroxybenzoyl group (C_7_H_6_O_3_) and a molecule of 3, 4, 5-trimethylcinnamyl group (C_12_H_16_O_5_). Based on this, *m/z* 443.1214 was caused by the loss of a molecule of C_12_H_16_O_5_ after CID. In addition, a higher response *m/z* 137.0248 fragment ion C_7_H_5_O_3_^−^ and *m/z* 239.0566 fragment ion C_12_H_15_O_5_^−^ were generated. The possible cleavage pathway of TenA is shown in Figure 3E. As shown in the TOF MS/MS spectrum, polygala oligosaccharide esters have similar characteristic neutral glucose or fructose residue loss, and the characteristic neutral loss is 162.0528. The representative compound polygala xanthones Ten was taken as an example. According to the MS/MS mass spectrum of SB (Figure 2F), the excimer ion peak was *m/z* 537.1284, and the main fragment ions of *m/z* 406.0690, 387.0737, 297.0414, and 243.0372 were generated in the secondary mass spectrum. Among them, *m/z* 406.0690 was caused by the loss of the pentose residues, and *m/z* 243.0372 was generated by further losing neutral glucose residues. Meanwhile, *m/z* 387.0737 was produced by the loss of 150.0528 characteristic neutral fragment ion and *m/z* 297.0414 was produced by the loss of the xanthone ring. The possible cleavage pathway of SB is shown in Figure 3F. As shown in the TOF MS/MS mass spectrum, polygala xanthones have similar characteristic residue loss, and the characteristic neutral loss is 132.0423, 150.0528, and 162.0528.

In summary, the compounds of *Panax ginseng* and polygala produced relatively abundant [M-H]^-^ or [M + HCOOH-H]^-^ excimer ion peaks in the negative ion mode. During cleavage, various substituents are sequentially removed to generate a series of less abundant fragment ions. If there are glycosyl substitutions on the parent nucleus, the glycosyls will be sequentially removed, and finally the parent nucleus aglycon fragment ions will be generated. In addition, each sugar group will also generate various sugar residue ions. Other ions produced during the cleavage process could also be used to aid diagnosis. Therefore, the possible diagnostic ions are summarized as shown in Table 1.

**TABLE 1:** Diagnostic ions for common compounds of the “ginseng–polygala” drug pair.

Parent nucleus structure	Diagnostic ions
PPD-type	m/z 783.4992 and m/z 621.4428
PPT-type	m/z 799.4928, m/z 637.4374, and m/z 475.3813
OLE-type	m/z 793.4468, m/z 475.3813, and m/z 455.3555
Polygala saponins	m/z 679.3759 and m/z 455.3189
Oligosaccharide esters	m/z 137.0248 and m/z 239.0566
Xanthones	m/z 297.0414 and m/z 315.0519

It provided excellent structural information through UHPLC-QTOF-MS analysis, and the complex representative compounds of GR + PR by diagnostic ions along with cleavage product ions were highlighted and analyzed.

#### 3.2.2 Molecular network analysis

**FIGURE 4:**
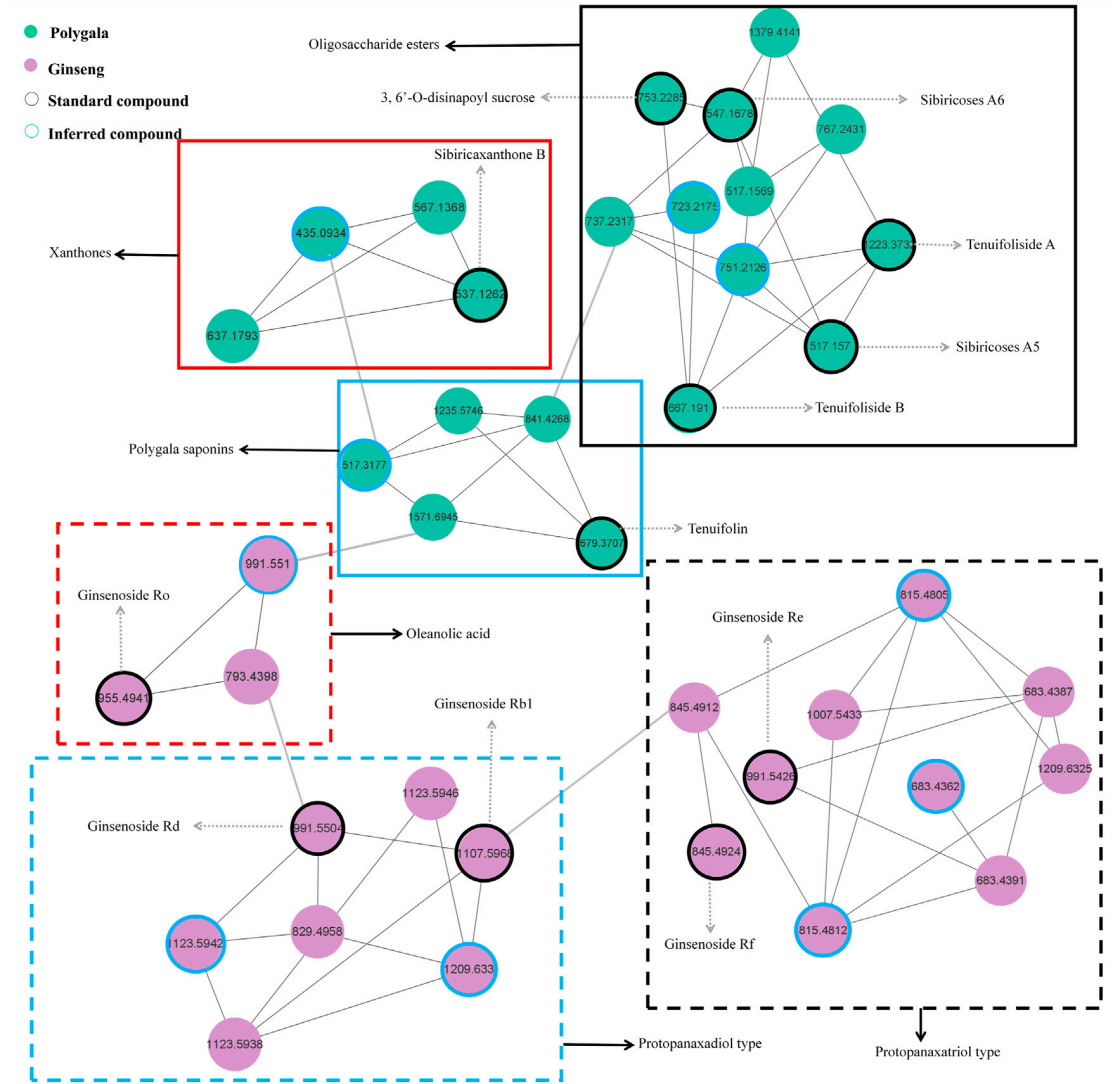
The molecular network of characteristic components in “ginseng–polygala” drug pair.

As shown in Figure 4, the network diagram was divided into six regions. The blue dotted area represents PPD-type ginsenosides, and the matched compound is consistent with GRd, which contains *m/z* 783.4992 and *m/z* 621.4428 fragment ions, and the reference substance GRb1 also appeared in this area. The black dotted area represents PPT-type ginsenosides, the matched compound is consistent with GRe, which contains *m/z* 799.4928 and *m/z* 637.4374 fragment ions, and the reference substance GRf also appeared in this area. The red dotted area represents OLE-type ginsenosides, and the matched compounds contain *m/z* 455.3555 oleanolic acid sapogenin fragment ions, and the reference substance GRo appeared in this area. The blue solid line area represents polygala saponins, the matched compounds all contain *m/z* 679.3759 and *m/z* 455.3189 characteristic fragment ions, and the standard substance Ten appeared in this area. The black solid line area represents polygala oligosaccharide esters, all matched compounds contain *m/z* 137.0248 fragment ion and neutral loss 162.0528, which is consistent with TenA. Meanwhile, the standard compounds A5, A6, TenB, and 3, 6′-O-ds also appeared in this area. The area of the red solid line represents polygala xanthones, and the matched compounds contain *m/z* 297.0414 xanthone ring characteristic ion, which is consistent with SB.

From the GR + PR extract MN diagram, we can find that each node represents a compound, and the different colors in the nodes represent samples from different sources. The connection between the nodes indicates that the two compounds have similar fragment ions. The closer the distance, the higher the similarity. The rapid identification and classification of compounds is facilitated by MN analysis. The network diagram also intuitively suggests the structure classification of some representative compounds and deserves further attention.

#### 3.2.3 Characteristic component analysis

**FIGURE 5:**
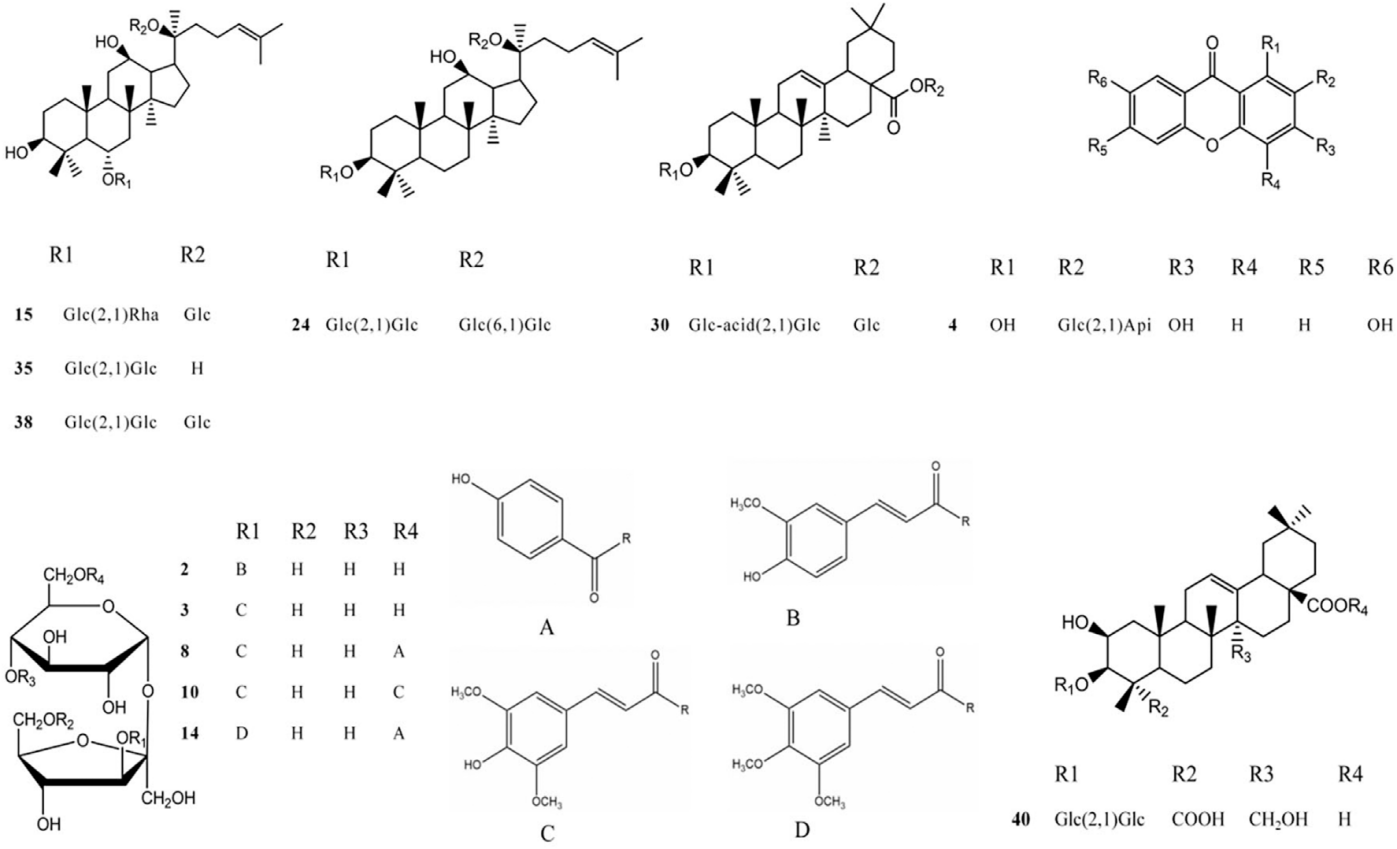
The structure of each component of different chemical structures. 2 A5; ***3*** A6; 4 SB; 8 TenB; 10 3,6′-O-ds; 14 TenA; 15 GRe; 24 GRb1; 30 GRo; 35 GRf; 38 GRd; and 40 Ten.

After the optimization of chromatographic separation conditions and MS detection parameters, a simple LC-QTOF-MS method was developed to detect the ingredients in GR + PR. The total ion chromatogram (TIC) of GR + PR is shown in Supplementary Figure S1. In the TIC, only a few peaks could be distinguished from the background, and most of the component ions with low abundance were submerged in the matrix-derived signals, leading to an easy miss of the interested components during the manual inspection. Therefore, the combination with database, reference comparison, diagnostic ion strategy, and MN was needed to identify the compounds, and 40 compounds were preliminarily identified. The molecular formulas, retention times, accurate molecular weights, fragment ion information, and structural types of the 40 compounds are shown in Table 2. Among them, 12 compounds, which are the representative active ingredients and, respectively, belonging to PPD-type (GRb1, GRd), PPT-type (GRe, GRf), OLE-type (GRo), polygala saponins (Ten), xanthones (SB), and oligosaccharide ester (A5, A6, TenB, 3,6′-O-ds, and TenA) category, were accurately identified in comparison with the reference substances. Their specific structures are shown in Figure 5. Furthermore, multiple studies have shown that the 12 compounds we selected showed potential benefit in neuroprotection and were particularly relevant for AD treatment (Li et al., 2015; Du et al., 2018; Li et al., 2019; Wang et al., 2019; Chen et al., 2020; Chen and Jia, 2020; Zhao et al., 2020; Li L. et al., 2021; Madhi et al., 2021; Wang et al., 2021; Yuan et al., 2021).

**TABLE 2:** Chemical composition analysis of the “ginseng–polygala” drug pair by UHPLC-QTOF-MS in the negative ion mode.

NO	Formula	tR (min)	Cal m/z	m/z	Δ(ppm)	[M-H]−or [M + HCOOH-H]−Fragment ions (m/z)	Compound	Type
1	C_22_H_30_O_14_	8.44	517.1563	517.1569	1.2	137.0223, 160.0107, and 175.0406	Arillatoses B	Oligosaccharide esters
2^a^	C_22_H_30_O_14_	9.44	517.1563	517.1570	1.4	137.0223, 160.0173, 175.0412, 193.0522, 341.1177, and 517.1604	Sibiricoses A5	Oligosaccharide esters
3^a^	C_23_H_32_O_15_	9.69	547.1668	547.1678	-0.7	137.0538, 175.0039, 179.0587, 190.0279, 205.0522, 223.0635, 341.1117, 367.1067, and 547.1737	Sibiricoses A6	Oligosaccharide esters
4^a^	C_24_H_26_O_14_	12.35	537.1250	537.1262	2.2	243.0372, 297.0414, 315.0517, 387.0737, 406.0900, and 537.1284	Sibiricaxanthone B	Xanthones
5	C_34_H_40_O_19_	12.47	751.2091	751.2126	4.6	397.0579, 412.0817, 427.1050, 443.1373, and 751.2163	Arillatoses A	Oligosaccharide esters
6	C_25_H_28_O_15_	12.79	567.1355	567.1368	2.1	272.0326, 297.0417, 315.0519, 345.0632, 357.0637, 399.0751, 417.0859, 447.0953, 549.1272, and 567.1402	Polgalaxanthone III	Xanthones
7	C_20_H_20_O_11_	12.94	435.0933	435.0934	0.1	243.0294, 272.0329, 300.0280, 315.0508, 330.0389, and 345.0613	7-O-methylmangiferin	Xanthones
8^a^	C_30_H_36_O_17_	13.35	667.1880	667.1910	4.5	137.0538, 175.0040, 190.0277, 205.0512, 223.0617, 239.0557, 281.0668, 461.1321, and 667.1940	Tenuifoliside B	Oligosaccharide esters
9	C_29_H_34_O_16_	13.89	637.1774	637.1793	2.9	443.1202, 461.1333, and 637.1857	Sibiricaxanthone F	Xanthones
10^a^	C_34_H_42_O_19_	14.82	753.2248	753.2285	4.9	137.0542, 175.0043, 190.0273, 205.0509, 223.0617, 265.0725, 325.0940, 367.1047, 529.1602, 547.1700, and 753.2323	3, 6′-O-disinapoyl sucrose	Oligosaccharide esters
11	C_33_H_40_O_18_	15.14	723.2142	723.2175	4.6	137.0168, 175.0402, 205.0512, 223.0617, 265.0727, 529.1597, 547.1704, and 723.2205	Arillanin A	Oligosaccharide esters
12	C_48_H_82_O_19_	15.28	1007.5421	1007.5453	3.2	475.3791, 637.4338, 799.4898, and 961.5449	20-Glc-ginsenoside Rf	PPT
13	C_42_H_72_O_14_	16.21	845.4893	845.4912	2.2	161.0455, 475.3820, 637.4375, and 799.4942	Ginsenoside Rg1	PPT
14^a^	C_31_H_38_O_17_	16.24	1223.3672	1223.3732	4.9	137.0248, 179.0358, 239.0566, 263.0570, 281.0675, 443.1214, and 681.2107	Tenuifoliside A	Oligosaccharide esters
15^a^	C_48_H_82_O_18_	17.24	991.5472	991.5426	4.6	161.0459, 179.0563, 475.3813, 619.4270, 637.4374, 765.4870, 783.4975, 799.4928, and 945.5529	Ginsenoside Re	PPT
16	C_35_H_44_O_19_	17.61	767.2404	767.2431	3.5	137.0526, 205.0510, 223.0618, 265.0725, 367.1043, 529.1589, and 767.2496	Tenuifoliside C	Oligosaccharide esters
17	C_34_H_42_O_18_	18.14	737.2298	737.2317	2.5	121.0297, 147.0457, 305.0892, 467.1427, 615.2023, and 737.2438	Reiniose A	Oligosaccharide esters
18	C_58_H_92_O_28_	18.79	1235.5702	1235.5746	4.4	137.0355, 337.1152, 425.3081, 455.3197, 469.1594, 555.1979, 1011.5315, 1205.5774, and 1235.5854	Arillatanoside A	Polygala saponins
19	C_62_H_76_O_35_	20.67	1379.4094	1379.4141	4.6	-	Tenuifoliose A	Oligosaccharide esters
20	C_41_H_70_O_13_	21.65	815.4788	815.4812	3.0	161.0462, 457.3706, 475.3821, 619.4274, 637.4383, and 769.4845	Ginsenoside F3	PPT
21	C_58_H_98_O_26_	22.06	1209.6274	1209.6325	4.2	621.4421, 783.4976, 945.5551, 1077.6001, and 1209.6475	Ginsenoside Ra1	PPT
22	C_42_H_66_O_17_	22.96	841.4227	841.4268	4.9	161.0463, 221.0663, 263.0773, 425.3071, 455.3171, and 841.4382	Reinioside A	Polygala saponins
23	C_41_H_70_O_13_	23.23	815.4788	815.4805	2.1	131.0346, 475.3814, 637.4372, 769.4837, and 815.4886	Ginsenoside F5	PPT
24^a^	C_54_H_92_O_23_	23.63	1107.5957	1107.5968	1.0	179.0569, 221.0676, 621.4431, 783.4993, 945.5548, and 1107.6101	Ginsenoside Rb1	PPD
25	C_36_H_62_O_9_	24.12	683.4365	683.4391	3.8	391.2862, 457.3720, 475.3812, and 637.4347	20S Ginsenoside Rh1	PPT
26	C_42_H_72_O_13_	24.29	829.4944	829.4958	1.6	161.0459, 375.2914, 459.3847, 621.4387, and 783.4943	Ginsenoside F2	PPD
27	C_58_H_98_O_26_	24.42	1209.6274	1209.6330	4.7	149.0468, 323.0994, 621.4400, 783.4999, 945.5554, 1047.5862, 1077.5993, and 1209.6489	Ginsenoside Ra2	PPD
28	C_53_H_90_O_22_	25.07	1123.5895	1123.5938	3.8	149.0452, 621.4389, 765.4833, 783.4935, 915.5386, 945.5481, and 1077.5908	Ginsenoside Rc	PPD
29	C_36_H_62_O_9_	25.28	683.4365	683.4387	3.2	161.0482, 353.1919, 391.2870, 475.3789, 553.3424, and 637.4368	20R Ginsenoside Rh1	PPT
30^a^	C_48_H_76_O_19_	25.35	955.4908	955.4941	3.4	455.3548, 523.3813, 569.3880, 613.3786, 731.4430, 793.4433, and 955.4980	Ginsenoside Ro	OLE
31	C_53_H_90_O_22_	25.73	1123.5895	1123.5946	4.5	783.4956, 945.5506, and 1077.5922	Ginsenoside Rb2	PPD
32	C_53_H_90_O_22_	26.11	1123.5895	1123.5942	4.2	783.4957, 945.5513, and 1077.5935	Ginsenoside Rb3	PPD
33	C_36_H_62_O_9_	27.18	683.4365	683.4362	-0.5	161.0483, 391.2846, 457.3703, 475.3816, and 637.4348	Ginsenoside F1	PPT
34	C_47_H_74_O_18_	28.07	991.5472	991.5510	3.8	161.0464, 459.3875, 621.4428, 765.4891, 783.4992, and 945.5546	Pseudoginsenoside RT1	OLE
35^a^	C_42_H_72_O_14_	28.28	845.4893	845.4924	3.7	161.0464, 475.3814, 637.4360, and 799.4923	Ginsenoside Rf	PPT
36	C_42_H_66_O_14_	28.88	793.4377	793.4398	2.3	455.3533, 569.3864, 613.3774, 631.3876, and 793.4420	Zingibroside R1	OLE
37	C_30_H_46_O_7_	30.27	517.3171	517.3177	1.3	351.2704, 367.3051, 407.2971, 411.2858, 425.3050, 469.2958, 487.3024, and 517.3221	Presenegenin	Polygala saponins
38^a^	C_48_H_82_O_18_	30.89	991.5472	991.5504	3.2	179.0558, 221.0667, 323.0985, 621.4398, 783.4962, and 945.5494	Ginsenoside Rd	PPD
39	C_75_H_112_O_35_	32.18	1571.6911	1571.6945	2.2	-	Onjisaponin B	Polygala saponins
40^a^	C_36_H_56_O_12_	33.08	679.3699	679.3707	1.2	425.3076, 455.3189, and 679.3759	Tenuifolin	Polygala saponins

^a^ Compared with references.

Thus, the combination of the MN strategy and UHPLC-QTOF-MS was used successfully for rapid, efficient, and comprehensive profiling of representative active ingredients in GR + PR, laying a foundation for the subsequent content determination of representative compounds in GR + PR. The developed strategy might accelerate and simplify operations for bioactive compound investigation in natural products. In addition, these experimental study results will serve as useful chemical fundamental information for improvements for drug (GR + PR) discovery work of AD treatment.

### 3.3 Content determination of related active components in the “ginseng–polygala” drug pair

**FIGURE 6:**
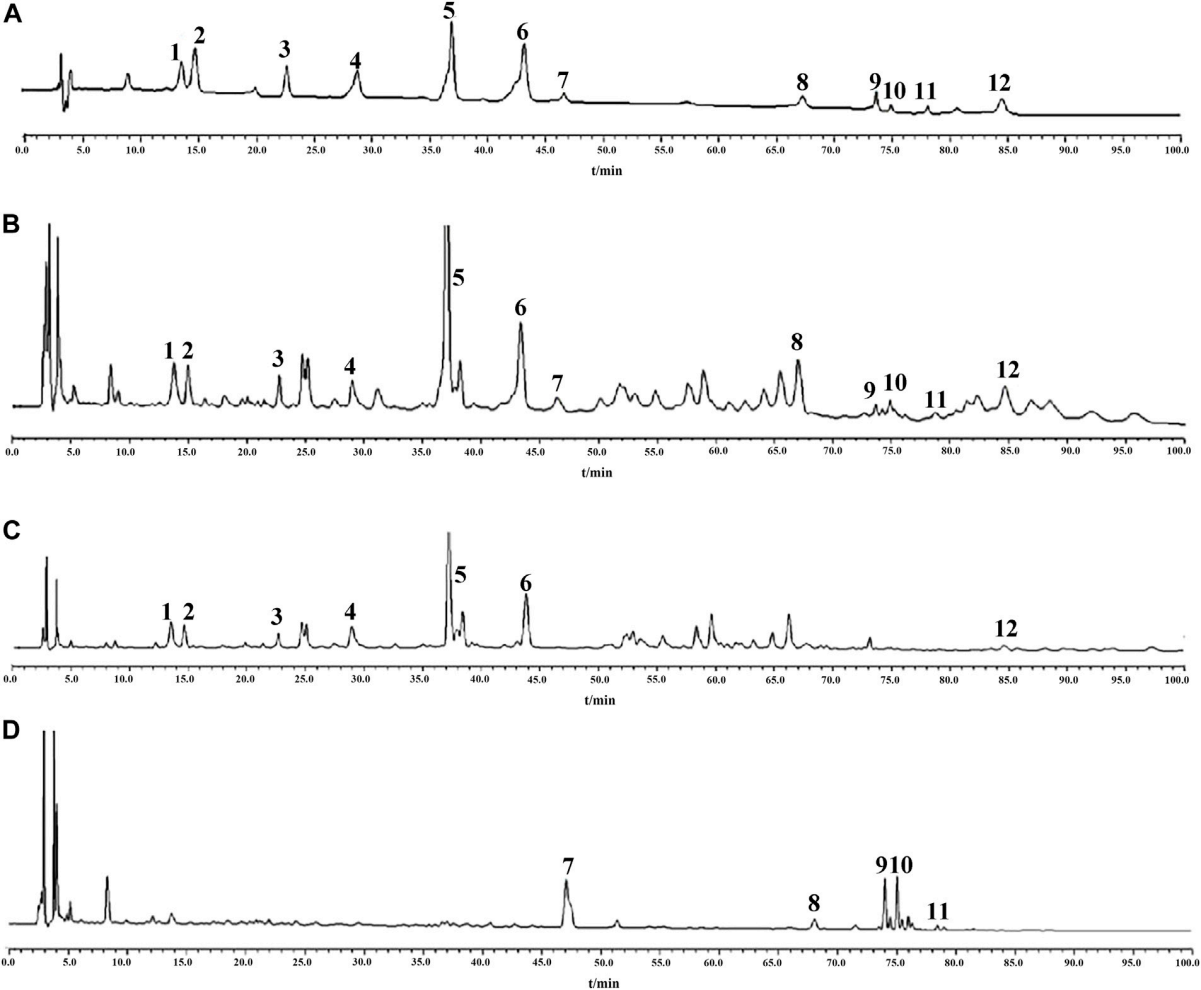
HPLC chromatogram of “ginseng–polygala” drug pair. Reference substances **(A)** GR + PR sample **(B)**, PR sample **(C)**, and GR sample **(D)**. 1 A5; 2 A6; 3 SB; 4 TenB; 5 3, 6′-O-ds; 6 TenA; 7 GRe; 8 GRb1; 9 GRo; 10 GRf; 11 GRd; and 12 Ten.

In order to explore the content of active ingredients in GR + PR, the typical HPLC was established. Quantitative analysis of 12 major bioactive compounds was validified. In addition, the content changes of the compounds before and after compatibility were compared to explore the effect of compatibility on the content of TCM. Also, the chromatograms of GR, PR, and GR + PR are shown in Figure 6. Compounds 1–12 compared with the reference represent A5, A6, SB, TenB, 3, 6′-O-ds, TenA, GRe, GRb1, GRo, GRf, GRd, and Ten, respectively. Simultaneously, GR solution has no chromatographic peaks in the corresponding positions of A5, A6, SB, TenB, 3,6′-O-ds, TenA, and Ten, and PR solution is also the same at the positions of GRe, GRf, GRb1, GRo, and GRd. There was no interference between the components, and the specificity of the method was good. Methodological validation including specificity, linearity and range, precision, stability, and accuracy (Supplementary Material) was carried out in the experiment to prove that the content determination method meets the testing requirements. The results show that the range of accuracy and precision were in line with the relevant standards, 12 components were completely separated, and the linear relationship was good, according to the method validation. The established method was accurate, repeatable, and stable, which provides a scientific basis for the establishment of the quality standard of GR + PR. Simultaneously, in this experiment, the orthogonal experimental method was used to optimize the optimal extraction process in GR + PR. On the basis of the research of Lee et al. Qi et al. (2012); Lee et al. (2016); Ji et al. (2020), the solvent dosage (50, 100, and 150 ml), mass fraction of methanol (50%, 70%, and 100%), and extraction time (30, 45, and 60 min) were taken as influencing factors. According to the L_9_ (3^3^) orthogonal test and variance analysis, the optimum extraction process was carried out when the amount of methanol was 150 ml, the mass fraction of methanol was 100%, and the extraction time was 60 min. The specific optimization procedure is shown in Supplementary Material.

Meanwhile, Table 3 shows the changes in the content of active ingredients before and after the combination using SPSS 17.0 statistical software. The results showed that the contents of GRf and GRd were significantly increased (*p* < 0.05), while the contents of SB, TenA, GRe, and GRo were significantly decreased (*p* < 0.05). It follows that after the compatibility of GR and PR, the content change of the components is not the simple addition of quantity but the comprehensive effect of the two medicines after the compatibility. The reason maybe including the pH value of the solution, the chemical reaction between the components, and thermal decomposition during the extraction process, leading to interaction between the components, which promotes or inhibits the dissolution of the active ingredient. Thereinto, extraction solvent and mutual dissolution of drug components play an important role as well (Wang and Ren, 2019). In addition, some studies have shown that some ginsenosides with similar chemical structures will undergo a certain degree of transformation during the extraction process (Shi et al., 2019). On one hand, the interaction between ginsenosides may be caused by the influence of temperature and extraction solvent. On the other hand, some acidic ginsenosides would split into acids, such as malonic acid and acetic acid, which affects the conversion process of saponins and eventually leads to content change (Gao et al., 2019; Chen et al., 2020). However, the specific mechanism of the content change of each component after compatibility is still unclear and needs further study.

**TABLE 3:** The contents of 12 components in the test solution (n = 3).

Compound	Content (mg·g^−1^)		
	GR	PR	GR + PR
A5	ND	1.874	1.761
A6	ND	1.078	1.050
SB	ND	1.947	1.332*
TenB	ND	1.228	1.203
3,6′-O-ds	ND	1.592	1.634
TenA	ND	0.461	0.333*
GRe	1.218	ND	1.104*
GRb1	5.320	ND	5.008
GRo	6.790	ND	5.196*
GRf	0.995	ND	1.091*
GRd	0.313	ND	0.494*
Ten	ND	0.864	0.767

Note: Compared with the content of each component in GR or PR, *p < 0.05; ND (not detected).

These results indicated that we can accurately and rapidly determine the content of active ingredients in GR + PR by HPLC and the change of content after compatibility may be one of the main reasons for the difference of efficacy. Moreover, quantitative analysis of bioactive compounds is helpful for quality evaluation and AD clinical utilization of GR + PR.

## 4 Conclusion

It is a very challenging task to study the pharmacodynamic effects of TCM and to characterize and identify the complex compounds. First of all, the regulatory effects of GR, PR, and their combination in oxidative stress, neuroinflammation, and cell activity were investigated through *in vitro* cell experiments, and the cell results showed that GR + PR has significant activity, which provided a basis for the evaluation of the efficacy of AD therapeutic drugs and the study of their mechanism of action. In the meanwhile, 40 chemical components were preliminarily identified and classified in the GR + PR through a combined UHPLC-QTOF-MS with MN strategy. Among them, 12 compounds were accurately identified in comparison with the reference substances. Furthermore, the content of these active components was simultaneously determined by HPLC. The foundation was laid for the active compound identification and quality control of GR + PR after the compatibility, and it provided new ideas for the research of TCM compounds in the treatment of AD. However, in order to further explore its underlying molecular mechanisms, in follow-up studies, we will reveal the scientific connotation of the compatibility from the perspectives of absorption, distribution, metabolism, and pharmacodynamics *in vivo*.

## Data Availability

The original contributions presented in the study are included in the article/Supplementary Material. Further inquiries can be directed to the corresponding authors.
