# Supplementary material for: Qualitative and quantitative analysis of the bioactive components of “ginseng–polygala” drug pair against PC12 cell injury based on UHPLC-QTOF-MS and HPLC
Source: Front Pharmacol. 2022 Dec 9;13:949757. doi: 10.3389/fphar.2022.949757 (PMC9780267; doi:10.3389/fphar.2022.949757)
Supplement: Supplementary file 1 [file DataSheet1.pdf]

Supplementary Material

1 Total ion chromatogram

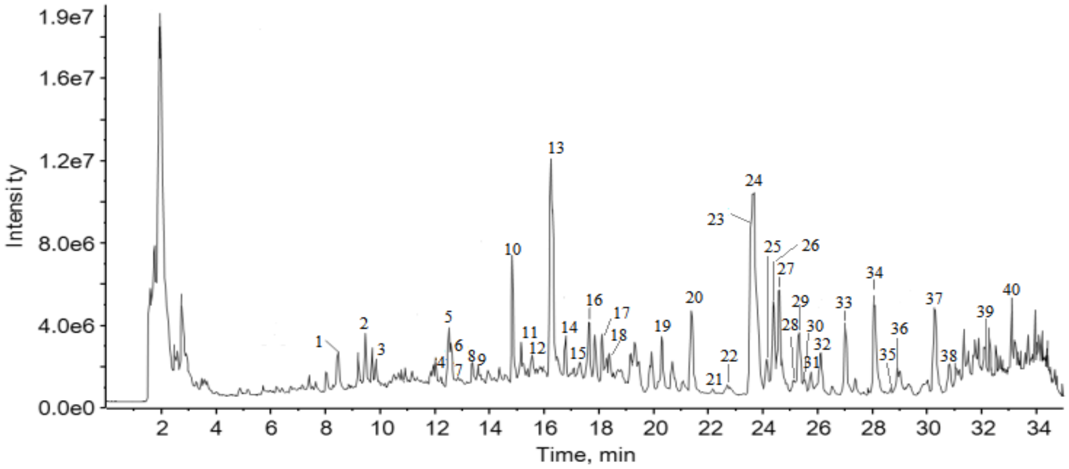

Figure S1. The total ion chromatogram of “ginseng-polygala” drug pair in the negative ion mode

2 Method validation of quantitative analysis

2.1 Specificity

The mixed reference solution and the sample solution were precisely measured, and the samples were injected according to the chromatographic conditions under “2.5”. The results showed that the retention times of the reference compounds (1)-(12) were 13.840, 14.948, 21.121, 28.967, 36.886, 43.002, 46.402, 67.154, 73.428, 74.761, 77.883 and 84.061 min, respectively, which were close to the real sample. And corresponding chromatographic peaks could be distinguished from the impurity peaks (R>1.5).

2.2 Linearity and range

To obtain the calibration curve, working solutions of six concentrations containing A5, A6, SB, TenB, 3,6’-O-ds, TenA, GRe, GRb1, GRo, GRf, GRd, and Ten were analyzed in triplicate. The calibration curves were established by plotting peak area versus the quantity of each analyte. In the regression equation Y = aX + b, X, Y and R^2 refer to the quantity of each substance, the peak area, and the correlation coefficient, respectively. Calibration curves of the twelve analytes showed good linearity in relatively wide dynamic ranges. The result was shown in Table S1.

Table S1 The regression equation of each component and its linear range

Compound	Retention time/min	Regression equation	Linear range/μg·mL^{-1}	R^2
A5	13.840	$Y=5.224\times10^3 X+5.191\times10^5$	54.00-1080	0.9996
A6	14.948	$Y=1.460\times10^4 X+6.692\times10^5$	28.00-560.0	0.9997
SB	21.121	$Y=2.365\times10^4 X+3.191\times10^5$	30.00-600.0	0.9995
TenB	28.967	$Y=4.715\times10^4 X+4.941\times10^5$	15.20-304.0	0.9996
3,6’-O-ds	36.886	$Y=6.346\times10^4 X-9.246\times10^4$	26.60-532.0	0.9991
TenA	43.002	$Y=2.063\times10^4 X+1.433\times10^5$	94.0-1880	0.9993
GRe	46.402	$Y=6.348\times10^3 X-6.405\times10^4$	37.50-750.0	0.9998
GRb1	67.154	$Y=2.728\times10^3 X-4.796\times10^4$	100.0-2000	0.9990
GRo	73.428	$Y=4.846\times10^3 X+4.866\times10^4$	14.00-345.0	0.9995
GRf	74.761	$Y=5.732\times10^3 X-1.443\times10^4$	49.80-996	0.9994
GRd	77.883	$Y=1.424\times10^3 X+2.615\times10^4$	86.0-1720	0.9994
Ten	84.061	$Y=2.024\times10^3 X+1.098\times10^5$	171.0-3410	0.9992

2.3 Precision

Take the same batch of ginseng-polygala respectively, prepare six solutions (40 mg/mL) in parallel according to the preparation method under “2.2”. Measure according to the chromatographic conditions under “2.5”, record the peak area of the twelve analytes in ginseng and polygala, and calculate the content of each component measured. The RSD for the twelve analytes were shown in Table S2, which demonstrated good precision for this method.

2.4 Stability

Take the solution of ginseng-polygala, then place them at room temperature for 0, 2, 4, 8, and 12 h respectively. Measure according to the chromatographic conditions under “7”, record the peak area of each component measured. All analytes were found to be stable within 12 h with RSD less than 2.0%, and the RSD for the twelve analytes were also shown in Table S2.

2.5 Accuracy

Precisely weighed six parts of ginseng and polygala powder with known content (40 mg/mL), put them in a stoppered conical flask. The reference substance containing A5, A6, SB, TenB, 3,6’-O-ds, TenA, GRe, GRb1, GRo, GRf, GRd, and Ten were added precisely, respectively. According to the preparation method under “2.2” prepare the sample solution. The sample was injected for HPLC measurement according to the chromatographic conditions under “2.5”, and the peak area of each reference substance chromatographic peak was calculated. Then, the external standard method was used to calculate the sample recovery. The average recoveries were calculated with the formula: recovery (%) = (amount found-original amount)/amount×100%. The results showed that the recovery rate of this method was good. The average recoveries and RSD of the twelve analytes were shown in Table S2, respectively.

Table S2 The result of method validation

Compound	Accuracy		Precision (RSD/%)	Stability (RSD/%)
	Average recoveries/%	RSD/%		
A5	95.7%	1.141%	1.262%	1.375%
A6	85.1%	0.868%	0.975%	1.996%
SB	116.7%	1.889%	1.293%	1.731%
TenB	113.5%	1.885%	0.7892%	1.005%
3,6’-O-ds	107.2%	1.941%	1.528%	0.943%
TenA	114.6%	1.900%	1.867%	0.3421%
GRe	109.9%	2.064%	1.148%	1.862%
GRb1	114.9%	2.075%	0.6298%	0.3389%
GRo	91.7%	1.665%	1.638%	1.969%
GRf	118.8%	1.400%	1.119%	1.963%
GRd	102.9%	1.507%	0.990%	2.079%
Ten	109.2%	1.599%	1.458%	0.3229%

3 Optimization of extraction methods

3.1 Orthogonal experimental results

Taking the amount of solvent (A), the mass fraction of methanol (B), and the extraction time (C) as influencing factors, three levels were selected in each factor. According to the research of Lee et al, specific values were as follows, the amount of solvent were 50, 100 and 150 mL; the mass fraction of solvents were 50% methanol, 70% methanol and 100% methanol; the extraction time were 30, 45 and

60 min. Taking the content of the index components as the evaluation index, the orthogonal experimental design was carried out with three factors and three levels, and the horizontal table of orthogonal test factors was shown in Table S3. The experiment was designed according to the $L_9(3^3)$ orthogonal table, and the extraction rate was calculated. The test results and analysis results were shown in Table S4, and the variance analysis result was shown in Table S5.

According to Table S4 and Table S5, among the three factors, the amount of solvent has the greatest influence on the extraction rate, and the effect of the mass fraction of methanol was the second. For the extraction time, its range was the smallest, the effect on the result was not significant. In addition, the solvent dosage was K3>K2>K1, the comparison of extraction time was K3>K1>K2, and the comparison result was K3>K2>K1, therefore, the optimal extraction process combination was: A3B3C3. That is, the optimum extraction process was when the amount of methanol was 150 mL, the mass fraction of methanol was 100%, and the extraction time was 60 min.

Table S3 Horizontal table of orthogonal test factors

Level	Factors		
	Solvent dosage (A)/mL	Mass fraction of methanol (B)/%	Extraction time (C)/min
1	50	50	30
2	100	70	45
3	150	100	60

Table S4 Results of orthogonal experiment

Number	A	B	C	GRe	3,6'-O-ds	Extraction rate/%	Comprehensive assessment indexes/%
**1**	1	1	1	0.47	0.87	17.44	45.91
**2**	1	2	3	0.34	0.89	17.64	42.28
**3**	1	3	2	0.36	0.94	21.29	47.03
**4**	2	1	3	0.40	0.99	19.81	47.89
**5**	2	2	2	0.55	1.00	20.56	53.53
**6**	2	3	1	0.41	1.01	18.43	47.36
**7**	3	1	2	0.91	1.27	20.04	69.42
**8**	3	2	1	1.17	1.44	22.75	83.14
**9**	3	3	3	1.25	2.24	21.10	98.55
**K1**	45.07	54.41	58.80				
**K2**	49.59	59.65	56.66				
**K3**	83.70	64.31	62.91				
**Range（R）**	38.63	9.9	6.25				

Table S5 Results of orthogonal experiment analysis of variance

Factors	Sum of squares of deviations	Degree of reedom	Variance	F-value	P-value
A	2676.199	2	1338.100	10.593	0.086
B	147.381	2	73.691	0.583	0.632
C	60.452	2	30.226	0.239	0.807
Error value	252.639	2	126.319		

3.2 Verification test

According to the optimal extraction process conditions obtained from the above experiment, three extraction experiments were carried out in parallel, and the comprehensive assessment result was used as indicator. The result was shown in Table S6, which showed that the process was stable and feasible.

Table S6 Validation of extraction method of “ginseng-polygala” drug pair

Reaction condition	Test number	Comprehensive assessment indexes/%
A3B3C3	1	89.74
	2	89.95
	3	89.15
	Average value	89.61
